# The Infectious Diseases BioBank at King's College London: archiving samples from patients infected with HIV to facilitate translational research

**DOI:** 10.1186/1742-4690-6-98

**Published:** 2009-11-03

**Authors:** Rachel Williams, Christine Mant, John Cason

**Affiliations:** 1 Department of Infectious Diseases, Guy's, King's College and St Thomas' School of Medicine, King's College London, 2nd Floor Borough Wing, Guy's Hospital, St Thomas' Street, London, SE1 9RT, UK; 2 The National Institute for Health Research, Biomedical Research Centre, Guy's and St Thomas' NHS Foundation Trust, UK

## Abstract

The King's College London (KCL) Infectious Diseases BioBank opened in 2007 and collects peripheral venous blood (PVB) from individuals infected with pathogens including human immunodeficiency virus (HIV). PVBs are fractionated into plasmas, lymphocytes and DNA and are then frozen. All donations are from subjects who have given 'open consent' so samples can be used for virtually any type of biomedical research. The HIV component of the BioBank contains samples from over 400 donations from 138 HIV+ patients. Thus, the KCL Infectious Diseases BioBank - together with establishments such as the Spanish HIV BioBank - is likely to expedite translational research into this infection.

## Commentary

A recent *Correspondence *described the Spanish HIV BioBank [1], and here we would like to draw the attention of the readers of *Retrovirology *to a similar initiative in London, the United Kingdom (UK). The fact that high-quality tissue collections from patients will undoubtedly play an important role in translational research led King's College London (KCL) to open an Infectious Diseases (ID) BioBank in 2007. Current interests of the KCL ID BioBank are pathogens of local and international importance, namely: HIV, hepatitis B virus and a variety of bacteria. This *Commentary *describes the structure and operation of the KCL ID BioBank, with particular reference to the HIV archive.

## Location and management of the BioBank

The BioBank is embedded within the KCL Department of Infectious Diseases and is affiliated to Guy's and St Thomas' Hospitals National Health Service (NHS) Trust which act as tissue collection centres (TCC). This NHS Trust is one of five flagship Academic Health Centres in the UK and hosts an NHS National Institute of Health Research BioMedical Research Centre. The location of the KCL ID BioBank is another key feature in addition to these outstanding academic and clinical credentials: firstly, its location permits the expansion of TCCs to other major London teaching Hospitals (a third TCC at King's College Hospital was opened in September 2009 and another at St George's Hospital is planned for 2010). Secondly, the local population is large (~7 million), diverse (~300 different languages are spoken), transient and suffers some of the highest HIV infection rates in Europe. Indeed, one percent of women attending ante-natal clinics at St Thomas' Hospital are HIV positive [2] and clinics at the three TCCs care for around 5,000 HIV+ patients.

Like the Spanish HIV tissue collection, the KCL ID BioBank has a director (JC), manager (RJW), and a governance committee comprising of scientists and clinicians. The functions of this committee are to ensure that the BioBank operates within current UK legislation, to make policy decisions, and to assess the scientific merit of research applications from academic or commercial investigators in Europe or the USA. It is also empowered to act as a local research ethics committee and provide ethical opinions for studies which apply to use BioBank samples.

## BioBank operation and sample archive

Research nurses at the TCCs identify patients of interest as well as hospital controls and inform them of the BioBank *via *information sheets. At their next routine visit, subjects are invited to participate. Healthy individuals are recruited *via *posters and are incentivized with gift vouchers. If subjects agree to participate, they sign consent forms which detail possible risks or discomforts associated with the procedures and the open-ended nature of the research that their samples may be used for. All subjects have the option to withdraw their permission and either leave their samples in the repository or have them destroyed. Consent forms are stored at the TCCs, and clinical samples together with patient data sheets are anonymised.

The BioBank has permission to collect two categories of clinical materials: (i) 'research samples' of PVB, urine and faeces and, (ii) 'residual samples', *i.e. *any excess tissues, biopsies, swabs or bodily fluids collected primarily for diagnostic purposes. To date, only PVB samples have been collected. Either type of sample can be collected from any patient (who is a conscious adult, but neither a prisoner nor mentally impaired) attending a routine clinical appointment and who is suffering from any infectious or inflammatory condition at any TCC registered by the BioBank at any NHS location in England, Wales or Northern Ireland.

Up to seventy-milliliters of PVB are collected from HIV+ patients into EDTA Vacutainors™ and are then sent by courier to the BioBank, together with anonymised clinical details. Unlike some tissue collections, all KCL ID BioBank samples are fractionated at one location by one dedicated team using one set of standardized operating procedures (SOPs) in a category III laboratory. PVBs are separated into plasmas (1 ml aliquots), viable peripheral blood mononuclear cells (PBMC: at 1 × 10^7^/vial), as well as cells for DNA extractions which comprise of whole PVB cell pellets (residual from plasma preparations: 1 ml aliquots) as well as granulocyte-rich cells (left over from PBMC isolations: 1 ml aliquots). All fractions are frozen to -80°C, and the PBMC samples are transferred to -196°C liquid nitrogen stores located nearby. The BioBank is equipped with a nucleic acid extraction robot and a laboratory for quality control testing.

**Figure 1:**
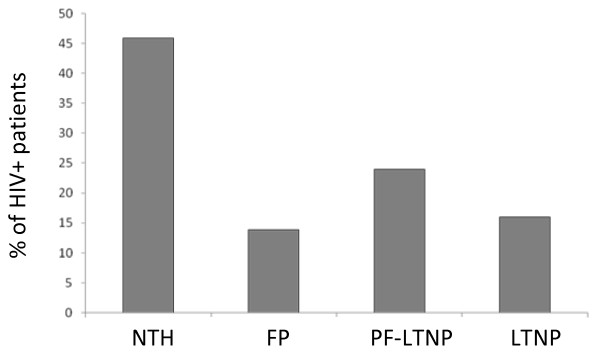
**Categories of HIV patients who have donated blood to the BioBank**. NTH: new to HAART, this cohort also includes some patients who would equally fall into the FP or LTNP categories; FP: fast progressor (<300 CD4+ cells/mm^3 ^within three years of their last negative HIV test); PF-LTNP: potential future LTNP; LTNP: long term non-progressors (which includes 10 patients with CD4+ cell counts of >500 cells/mm^3 ^in eight years of follow-up and 3 'elite' LTNP with CD4 cell counts of >600 cells/mm3 for ten years of follow-up [3].

To date, the BioBank has collected PVB from 17 healthy subjects (as of September 2009, n = 51 sample donations) and 60 non-infected hospital controls (60 donations), 80 patients with hepatitis B (80 donations), 185 patients with bacteraemias (185 donations), as well as from 143 patients with HIV (410 donations). TCCs recruit longitudinal samples from HIV patients with interesting or unusual clinical histories, specifically: (i) patients before and after the initiation of highly active anti-retroviral therapy (HAART),'new to HAART' (NTH); (ii) 'fast progressors' (FP), defined as patients with <300 CD4+ cells/mm^3 ^within four years of diagnosis; (iii) 'potential future long-term non-progressors' (PF-LTNP: those with undetectable or low viral loads, stable CD4+ cell numbers, long periods of clinical latency *etc.*); and (iv) 'long-term non-progressors' (LTNP) who fulfill the criteria of Grabar *et al. *2009 [3]. The BioBank holds samples from ten (7.2%) LTNP patients, and three (2.1%) 'elite' LTNP (Figure 1). For comparison, rates of LTNP and elite LTNP amongst HIV+ patients in France are 0.4% and 0.05% respectively [3]. For the groups (iii) and (iv), patients who have never received any anti-retroviral therapy are prioritized for recruitment. Some patients can fall into two classifications (*e.g. *~50% of fast progressors are about to receive HAART when recruited). Longitudinal samples are collected from all HIV patients This is an ongoing process. All samples are taken when patients attend their routine clinical appointment, and the frequency differs between groups - NTH patients provide one or two pre-treatment samples, then others at one, four, seven and twelve months after starting therapy. Other groups donate samples at six monthly intervals.

In addition to archiving biological samples, the BioBank maintains a database of clinical information on HIV donors which includes: histories of CD4+ cell numbers and plasma viral loads; dates of birth, last known HIV negative result and first positive HIV test; ethnic origin; gender; HAART received; and, any complicating infections. The database also contains sample processing information (dates and times of: venepuncture, processing and freezing) and details of numbers of aliquots stored or transferred to researchers.

## Distribution of samples

Researchers wishing to use BioBank samples are provided with an information pack and must obtain a positive ethical opinion and project approval by the governance committee. During this process they must specify the number and types of samples which will be needed for their study. BioBank samples are donated under 'open consent' so that virtually any type of immunological, genetic or microbiological testing can be performed on them. Exclusions to this are studies which involve: (i) testing of safety of cosmetics or consumer products; (ii) animal research; (iii) investigations into the termination of pregnancy or reproductive cloning; and, (iv) stem cells. There are no commercial restrictions on the use of BioBank samples (*e.g. *BioBank materials can be used to produce commercial products by cloning).

After approval, UK researchers must sign a materials transfer agreement (MTA) and the BioBank will then provide samples together with a file containing the technical and clinical information held in the database. An MTA suitable for International collaborations which meets U.S.A. and European governmental standards is currently being prepared. Until present all such samples have been provided pro bono; however, a discretionary charge to subsidize processing and storage costs will be introduced; and it is anticipated that this will be incorporated into researchers' grant applications. The types of studies currently using the BioBank include: investigations of DNA polymorphisms of the restriction factor tetherin, the viral permissivity factor ps20, functional studies of regulatory T-cells, and HIV sequencing studies.

## Quality control

A major challenge for all BioBanks is the continuous improvement of quality-control measures. For example, reliable RNA isolation was identified by the governance committee of being of importance; thus, the PAXgene™ system is being introduced to stabilize PVB RNA at venepuncture. More generally, standardization of SOPs for processing biopsies [4,5] has led to the establishment of the TuBaFrost association of European cancer tissue-banks [6]. Such harmonization needs to be extended to accommodate differing national legal and ethical regulations [7]. Thus, the KCL ID BioBank is a member of the International Society for Biological and Environmental Repositories and has based its SOPs upon UNE-EN-ISO 9001:2000 in order to facilitate future inter-biobank networking capabilities.

Pre-analytical variation is a major source of experimental error [8] and a major area of concern for biobanks is to ensure that archived material does not become degraded over time. PVBs are collected from HIV+ patients and processed within a collection-to-freezer window of <24 hours and a target of freezing >75% of PVB samples within 4 hours of venepuncture. All freezers are alarmed and checked routinely for temperature fluctuations. Plasma and PBMC samples released to researchers have not undergone a freeze-thaw cycle and all DNA samples are quantified and tested for the absence of polymerase chain reaction (PCR) inhibitors (by amplification of the human β-globin gene).

Random samples are also tested for the presence of viral nucleic acids by PCR (PVB DNA) and reverse-transcription-PCR (plasma) combined with cloning and sequencing. This has enabled the BioBank to establish a library of >500 cloned and sequenced near full-length (1490 bp) HIV-1 clade B *gag *genes, which are also available to external researchers.

A subset of the KCL ID BioBank collection does not conform to the quality-control measures described above. These include historic samples such as 1,000 HIV+ plasmas collected in the 1990s which are of unknown provenance in terms of the number of freeze-thaw cycles they have undergone. These are stored separately from the main BioBank, and methods for assessing their integrity are being explored. Plasma concentrations of soluble CD40 can be useful biomarkers in this respect, but cannot be used for HIV+ samples [9]; thus, plasma hormone levels [10] are being investigated for this purpose.

## Growth of the archive and future challenges

Whilst no single performance indicator can adequately summarize the growth of the KCL ID BioBank over the past two years, incidental statistics can be informative. In each of the first two quarters of 2007, there were around fifteen HIV+ patient visits *per *quarter whereas in the same quarters of 2009 they were about sixty visits.

Pressing challenges which the BioBank will need to address in the near future include: (i) data management, as researchers are bound by the MTA to provide experimental data back to the BioBank. This will lead to the exciting possibility of multivariate analyses in silica across a range of laboratory measurements of our HIV+ patients; (ii) integration of the BioBank electronic database with the bar-coded sample tubes via scanners: this will permit the automated updating of records; and (iii) increasing staffing levels.

## Conclusion

This *Commentary *provides an overview of the KCL ID BioBank. One additional significant property of this facility which should be highlighted is the high-degree of flexibility engineered into the ethical permissions. This means that it can be reactive to the emergence of new pathogens. Plans are now being made for the collection of samples from the anticipated second wave of the H1N1 ('swine') influenza virus infections. In conclusion, whilst the KCL ID BioBank currently holds fewer HIV samples than its Spanish counterpart, it is an important and rapidly growing resource which will undoubtedly expedite translational research of pathogens in the early part of the 21^st ^century.

## Competing interests

The authors declare that they have no competing interests.

## Authors' contributions

JC is the KCL BioBank director and, together with the BioBank manager (RJW), is responsible for the development of protocols and quality management. CM contributed to quality control, protocol development and is responsible for managing the equipment. All authors contributed intellectually to the writing of this article.

## References

[B1] García-Merino I de Las Cuevas N Jiménez JL Gallego J Gómez C Prieto C Serramía MJ Lorente R Muñoz-Fernández MA The Spanish HIV BioBank: a model of cooperative HIV research Retrovirology 2009 6 27 19272145 10.1186/1742-4690-6-27 PMC2667474

[B2] Health Protection Agency http://www.hpa.org.uk/web/HPAwebFile/HPAweb_C/1194947327144

[B3] Grabar S Selinger-Leneman H Abgrall S Pialoux G Weiss L Costagliola D Prevalence and comparative characteristics of long-term nonprogressors and HIV controller patients in the French Hospital Database on HIV AIDS 2009 23 1163 9 10.1097/QAD.0b013e32832b44c8 19444075

[B4] Knox K Kerr DJ Establishing a national tissue bank for surgically harvested cancer tissue Br J Surg 2004 91 134 136 10.1002/bjs.4486 14760657

[B5] Mager SR Oomen MH Morente MM Ratcliffe C Knox K Kerr DJ Pezzella F Riegman PH Standard operating procedure for the collection of fresh frozen tissue samples Eur J Cancer 2007 43 828 834 10.1016/j.ejca.2007.01.002 17329097

[B6] Isabelle M Teodorovic I Morente MM Jaminé D Passioukov A Lejeune S Therasse P Dinjens WN Oosterhuis JW Lam KH Oomen MH Spatz A Ratcliffe C Knox K Mager R Kerr D Pezzella F Vijver M van de van Boven H Alonso S Kerjaschki D Pammer J Lopez-Guerrero JA Llombart Bosch A Carbone A Gloghini A van Veen EB van Damme B Riegman PH TuBaFrost 5: Multifunctional central database application for a European tumour bank Eur J Cancer 2006 42 3101 3109 10.1016/j.ejca.2006.04.032 17029787

[B7] Riegman PHJ Dinjens WNM Oosterhuis JW Biobanking for interdisciplinary clinical research Pathobiology 2007 74 239 244 10.1159/000104451 17709966

[B8] Carraro P Plebani M Errors in a stat laboratory: types and frequencies 10 years later Clin Chem 2007 53 1338 42 10.1373/clinchem.2007.088344 17525103

[B9] Langelle J Panopoulos E Betsou F Soluble CD40 ligand as a biomarker for storage-related preanalytic variations of human serum Cytokine 2008 44 275 282 10.1016/j.cyto.2008.08.010 18851919

[B10] Evans MJ Livesy JH Ellis JM Yandle TG Effect of anticoagulants and storage temperature on stability of plasma and serum hormones Clin Biochem 2001 34 109 112 10.1016/S0009-9120(01)00196-5 11311219

